# Comparative Study of the Orientation and Order Effects on the Thermoelectric Performance of 2D and 3D Perovskites

**DOI:** 10.3390/nano14050446

**Published:** 2024-02-28

**Authors:** Yi-Hsiang Wang, Cheng-Hsien Yeh, I-Ta Hsieh, Po-Yu Yang, Yuan-Wen Hsiao, Hsuan-Ta Wu, Chun-Wei Pao, Chuan-Feng Shih

**Affiliations:** 1 Department of Electrical Engineering, National Cheng Kung University, Tainan 70101, Taiwan; s106064029@gmail.com (Y.-H.W.); n28084012@mail.ncku.edu.tw (C.-H.Y.); n28064012@mail.ncku.edu.tw (Y.-W.H.); 2 Research Center for Applied Sciences, Academia Sinica, Taipei 11529, Taiwan; arda800411@gmail.com (I.-T.H.); winniebird0519@gmail.com (P.-Y.Y.); 3 Department and Institute of Electrical Engineering, Minghsin University of Science and Technology, Hsinchu 30401, Taiwan; htwu@must.edu.tw; 4 Applied High Entropy Technology (AHET) Center, National Cheng Kung University, Tainan 70101, Taiwan; 5 Hierarchical Green-Energy Materials (Hi-GEM) Research Center, National Cheng Kung University, Tainan 70101, Taiwan

**Keywords:** perovskite, thermoelectric materials, 2D and 3D perovskites, non-equilibrium molecular dynamics (NEMD), thermal conductivity

## Abstract

Calcium titanium oxide has emerged as a highly promising material for optoelectronic devices, with recent studies suggesting its potential for favorable thermoelectric properties. However, current experimental observations indicate a low thermoelectric performance, with a significant gap between these observations and theoretical predictions. Therefore, this study employs a combined approach of experiments and simulations to thoroughly investigate the impact of structural and directional differences on the thermoelectric properties of two-dimensional (2D) and three-dimensional (3D) metal halide perovskites. Two-dimensional (2D) and three-dimensional (3D) metal halide perovskites constitute the focus of examination in this study, where an in-depth exploration of their thermoelectric properties is conducted via a comprehensive methodology incorporating simulations and experimental analyses. The non-equilibrium molecular dynamics simulation (NEMD) was utilized to calculate the thermal conductivity of the perovskite material. Thermal conductivities along both in-plane and out-plane directions of 2D perovskite were computed. The NEMD simulation results show that the thermal conductivity of the 3D perovskite is approximately 0.443 W/mK, while the thermal conductivities of the parallel and vertical oriented 2D perovskites increase with *n* and range from 0.158 W/mK to 0.215 W/mK and 0.289 W/mK to 0.309 W/mK, respectively. Hence, the thermal conductivity of the 2D perovskites is noticeably lower than the 3D ones. Furthermore, the parallel oriented 2D perovskites exhibit more effective blocking of heat transfer behavior than the perpendicular oriented ones. The experimental results reveal that the Seebeck coefficient of the 2D perovskites reaches 3.79 × 10^2^ µV/K. However, the electrical conductivity of the 2D perovskites is only 4.55 × 10^−5^ S/cm, which is one order of magnitude lower than that of the 3D perovskites. Consequently, the calculated thermoelectric figure of merit for the 2D perovskites is approximately 1.41 × 10^−7^, slightly lower than that of the 3D perovskites.

## 1. Introduction

Thermoelectric materials convert heat energy into electrical energy, making them suitable for applications such as waste heat recovery in factories or automobiles. A desirable characteristic of a thermoelectric material is to possess both high electrical conductivity and low thermal conductivity, creating a unique material structure known as a phonon glass electron crystal [1,2,3,4]. This structure resembles that of a glass material, which is not efficient in conducting heat, combined with a crystalline structure that facilitates electrical conduction. It is this combination that enables the material to achieve the maximum value of its thermoelectric figure of merit (ZT), thus maximizing its thermoelectric efficiency.

In thermoelectric materials, the Seebeck coefficient (*S*) represents the ability of a material to generate a voltage when subjected to a temperature gradient. The electrical conductivity (*σ*) indicates how well the material conducts electricity, while the thermal conductivity (*k*) represents its ability to conduct heat. The performance of thermoelectric materials is typically characterized by their thermoelectric figure of merit (ZT), which combines these properties and is given by the equation,

(1) $$\mathrm{ZT}=\frac{S^{2}\sigma }{k}T$$

In practice, materials that exhibit good electrical conductivity often also have high thermal conductivity, while insulating materials have low thermal conductivity but extremely poor electrical conductivity. Therefore, research in the field of thermoelectrics primarily focuses on inorganic semiconductor materials, as they have the potential to balance both electrical and thermal properties to achieve high ZT values.

Organic metal halide perovskites exhibit excellent optoelectronic properties, including high light absorption coefficients and carrier mobility. These properties make them suitable for applications in fields such as solar cells [5,6,7], photodetectors [8], and light-emitting diodes [9,10,11,12,13,14,15]. While metal halide perovskites typically have a 3D structure [16], they can also form 2D layered structures known as Ruddlesden–Popper phases [17,18,19]. These 2D phases are represented by the chemical formula (R-NH_3_)_2_A_n−1_B_n_X_3n+1_, where R-NH_3_ represents an organic long-chain or cyclic ammonium cation. Examples of R-NH_3_ include butylammonium (BA^+^) with four carbon atoms and phenethylammonium (PEA^+^) with a benzene ring structure. These organic cations act as spacers between the layers, while the remaining composition is the same as the 3D perovskite structure [20,21]. In the 2D perovskites, the parameter *n* refers to the number of octahedral layers in the perovskite crystal. For example, when *n* = 1, it represents a structure with one octahedral layer surrounded by R-NH_3_ cations on both ends. When *n* = 2, there are two layers, and so on. Experimental studies have demonstrated the formation of 2D perovskites with different values of *n* by adjusting the chemical molar ratios of R-NH_3_, AX, and BX_2_ precursors [22].

Two-dimensional perovskites can be classified into different orientations: parallel (horizontal) or perpendicular (vertical) orientations, depending on the arrangement of their crystal structure [23]. When *n* ≤ 2, the perovskites tend to grow with a preferred parallel orientation, with the crystal structure aligned along the [010] direction. On the other hand, when *n* > 2, the preferred orientation is perpendicular, with the crystal structure aligned along the (111) direction. These different crystal orientations in 2D perovskites result in variations in their optoelectronic properties.

In the case of the parallel orientation, where the organic long-chain cations act as barriers on the top and bottom, the transport of carriers from the bottom layer to the top electrode is impeded. As a result, when these materials are used in solar cell devices, the perpendicular orientation is preferred, as it allows for better carrier transport and performance [24].

In recent years, the application of perovskite materials in the field of thermoelectrics has emerged as a promising research direction. This is because perovskites exhibit the characteristics of a phonon glass electron crystal, where the inorganic octahedral structure provides channels for carrier transport while the organic cations disrupt phonon transport. The presence of the organic cations leads to higher phonon scattering rates and shorter phonon mean free paths, effectively reducing the thermal conductivity. This property helps maintain a temperature gradient between the hot and cold ends, thereby improving the thermoelectric conversion efficiency [25,26]. In 2016, Z. Guo et al. [27] measured the thermal conductivity of MAPbI_3_ thin films using time-domain thermo-reflectance techniques and confirmed that its thermal conductivity is only 0.5 W/m-K. In 2016, A. Filippetti et al. [28] used ab initio calculations and scattering rate models to study the electrical conductivity, Seebeck coefficient, and thermal conductivity of MAPbI_3_ in various structures, including single crystals and polycrystals. The computational results showed that although the power factor ($S^{2}\sigma$) of MAPbI_3_ is not particularly outstanding compared to other traditional thermoelectric materials, its extremely low thermal conductivity significantly enhances the overall thermoelectric figure of merit. This indicates the potential practical application of MAPbI_3_ in the field of thermoelectrics. In 2019, Y. Xiong et al. [29] conducted a series of measurements on MAPbI_3_ films to determine their thermoelectric properties. They found that doping a small amount of bismuth into the MAPbI_3_ film improved the surface morphology, resulting in an increased Seebeck coefficient and electrical conductivity. The results showed that the power factor of the MAPbI_3_ film doped with a small amount of bismuth was significantly enhanced by three orders of magnitude compared to the undoped MAPbI_3_ film. In 2021, S. J. Yang et al. [30] doped a small amount of MACl into the 2D perovskite (PEA)_2_(MA)_n−1_Sn_n_I_3n+1_ to reduce phase separation. Phase separation refers to the simultaneous presence of 2D perovskites with different values of *n* during the fabrication process. Through photoexcitation spectroscopy analysis, it was confirmed that doping a small amount of MACl resulted in a single *n*-value distribution of the 2D perovskite, reducing the boundary defects between different types of perovskites and thereby improving the carrier mobility and electrical conductivity of the material.

Currently, there is still a lack of research on the influence of the orientation of 2D perovskites on their thermoelectric properties. Additionally, the thermal conductivity of perovskites is difficult to measure. Therefore, only rough estimates of thermal conductivity around 0.5 W/m-K are used to calculate the thermoelectric figure of merit in literature, regardless of the orientation and order of the 2D materials. In this study, a series of non-equilibrium molecular dynamics (NEMD) simulations are employed to calculate the thermal conductivity of 2D perovskite films with different orientations. Experimental growth of 2D perovskite films with various *n*-values is performed, and the measured electrical conductivity and Seebeck coefficient are used to calculate the thermoelectric figure of merit of the perovskite material. These research findings will provide important insights for future researchers in the field of perovskite thermoelectric materials.

## 2. Materials and Methods

### 2.1. Non-Equilibrium Molecular Dynamics Simulations

**Figure 1:**
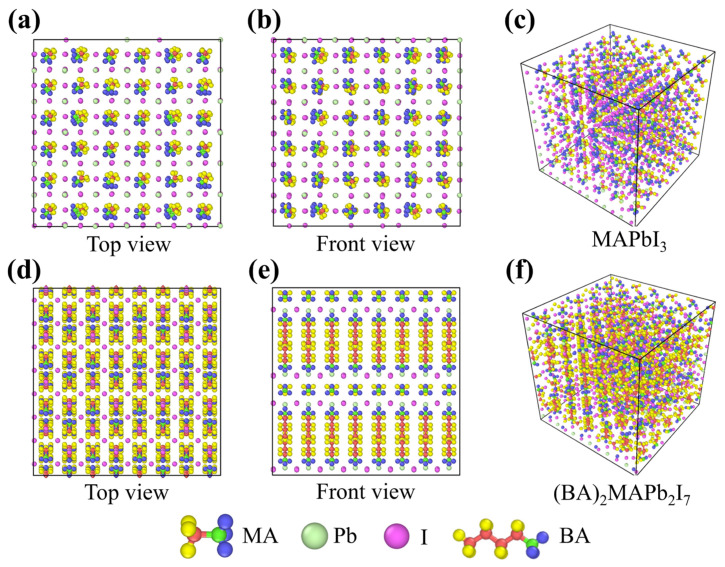
The initial model structure of (**a**–**c**) 3D perovskite and (**d**–**f**) 2D perovskite.

**Figure 2:**
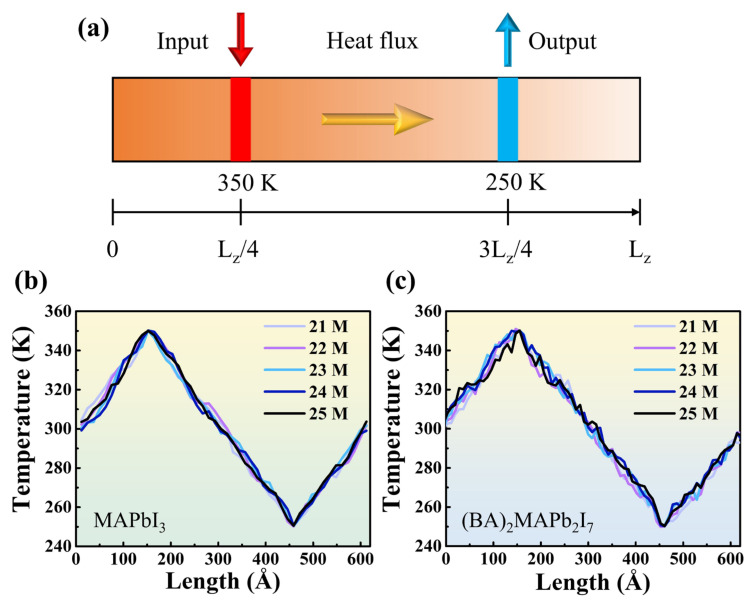
(**a**) Schematic diagram of non-equilibrium molecular dynamics (NEMD) hot/cold regions. Simulation of the temperature distribution from step 21,000,000 to step 25,000,000, (**b**) 3D perovskite, (**c**) 2D perovskite.

The initial atomistic structures of 3D and 2D perovskite are displayed in Figure 1. To describe the interatomic interactions in molecular dynamics simulations, we employed the empirical potentials by A. Mattoni [31] and by M. B. Fridriksson [32] to describe the interatomic interactions of 3D perovskite (MAPbI_3_) and 2D perovskite (BA)_2_MA_n−1_Pb_n_I_3n+1_, respectively. Due to the ideal lattice structure arrangement of atoms in the perovskite model at 0 K in the literature, which does not represent the actual atomic arrangement in a 300 K environment, and considering that the initial velocities do not conform to the Maxwell–Boltzmann distribution at 300 K, the NPT simulation was initially conducted using the Nosé–Hoover thermostat to achieve a balanced state where the system’s temperature and velocities approach around 300 K. In Figure S1, the 3D and 2D perovskite structures are compared under the same size, illustrating the changes in system temperature and potential energy over time during the equilibrium molecular dynamics simulation. From the potential energy plot, it can be observed that the potential energy quickly reaches equilibrium and oscillates around a certain value. The red line represents the final average potential energy over 22 million steps, indicating that setting the equilibrium molecular dynamics steps to 11 million is sufficient for the system to reach a steady state. The thermal conductivities of 2D and 3D perovskites were computed by a series of non-equilibrium molecular dynamics simulations. The simulation cell was firstly equilibrated in the NPT ensemble, namely, the number of atoms, cell pressure, and cell temperature is kept constant during simulation. In the present study, we performed a total number of five million steps to equilibrate the 2D or 3D perovskites, and the pressure and temperature were set to 1 atm and 300 K, respectively. After the simulation cell was equilibrated, we switched to the NVE ensemble for fifteen million steps for subsequent NEMD simulations. To perform NEMD simulations, hot and cold regions located at L_z_/4 and 3L_z_/4 of the simulation cell, respectively, were created. During the NEMD simulations, the temperatures at the hot/cold region were fixed at 350/250 K, respectively, by imposing the Langevin thermostat to the NVE ensemble to create the temperature gradient. The simulation cell settings for the NEMD simulations are depicted in Figure 2a. During the transition from NPT (constant Number of particles, Pressure, and Temperature) conditions to NVE (constant Number of particles, Volume, and Energy) conditions, the system undergoes some changes in physical properties because the control method shifts from temperature control to total energy control. Therefore, it is necessary to simulate the system under NVE conditions for a period to reach a new steady state before proceeding with subsequent data analysis. The overall simulation process involves 10 million steps of equilibrium molecular dynamics under NPT conditions, followed by the initiation of non-equilibrium molecular dynamics under NVE conditions for 15 million steps, totaling 25 million steps. The last 5 million steps are used for data analysis, and the time step size is set at 0.5 fs. In Figure 2b,c, temperature distribution graphs from steps 20 million to 25 million during the NVE simulation are presented. The average temperature of the thin film is output every 10 million steps, resulting in five temperature distribution curves. It can be observed that these five curves highly overlap, and the overall temperature standard deviation is within 5 K. This confirms that the temperature differences in the last 5 million steps are minimal, indicating that the system’s temperature has reached a converged state. This also signifies that the 15 million steps of non-equilibrium molecular dynamics are sufficient for the system to achieve a steady state.

The energy flux in/out of the hot/cold region owing to the imposed Langevin thermostat was computed and recorded as the heat flux *q* across the simulation cell. The thermal conductivity was subsequently computed using the Fourier law in Equation (2):

(2) $$\vec{q}=-k\frac{dT}{dx}$$

where *q* represents the heat flux, *k* denotes the thermal conductivity, and *dT/dx* represents the temperature gradient. Note that the time step during both the cell equilibration stage (NPT simulations) and subsequent NEMD stage were set to 0.5 fs. In order to eliminate the finite size effects of NEMD simulations originated from the limited excited phonon vibration modes due to finite simulation cell length, a series of NEMD simulations of different simulation cell lengths were performed and the respective thermal conductivities were computed. Then, the thermal conductivity of the bulk perovskite (namely, infinite cell length) can be extracted by extrapolating the thermal conductivities as the function of the inverse of the cell length to zero.

During the execution of NEMD, phonon transfer near the hot/cold regions is influenced by the energy influx and efflux, resulting in a nonlinear temperature distribution. When sampling, to avoid the nonlinear region, an additional removal of temperature samples based on a temperature-dependent thermal conductivity cutoff is applied, as illustrated in Figure S2b,d,f for 3D perovskite. A cutoff of 0 signifies that all temperature data within the range of 350 K to 250 K are selected for thermal conductivity calculation, as shown in Figure S2a. Cutoff 4 means that four temperature data points at both ends within the range of 350 K to 250 K are excluded, and the thermal conductivity is calculated based on the distribution of the remaining temperatures, as depicted in Figure S2c. Cutoff 8 indicates the exclusion of eight temperature data points at both ends within the range of 350 K to 250 K, and the thermal conductivity is calculated based on the distribution of the remaining temperatures, as shown in Figure S2e. A higher cutoff number implies a narrower range of temperatures selected for thermal conductivity calculation. To enhance the accuracy of thermal conductivity calculations, a certain number of temperature sampling ranges will be excluded. On the other hand, when the temperature distribution graph shows a highly linear distribution, the relationship between thermal conductivity and cutoff tends to be closer to a horizontal line, resulting in more accurate subsequent thermal conductivity calculations. Conversely, when the temperature distribution graph exhibits a low degree of linearity, the relationship between thermal conductivity and cutoff will have larger variations, leading to larger errors in subsequent thermal conductivity calculations. A similar approach for the 2D perovskite is plotted in Figure S3.

### 2.2. Preparation of Perovskite Materials

The ITO glass substrate is immersed in acetone, isopropanol, and deionized water and sonicated for 15 min using an ultrasonic oscillator to remove dust and grease adhered to the substrate. The ITO glass substrate is then cut into a size of 2 cm × 2 cm and dried using a nitrogen gun. Finally, it is placed in a 50 °C oven for drying. UV-Ozone treatment is performed for 20 min to clean the surface and remove organic residues. Subsequent processes are conducted inside a low-oxygen glove box environment (<1 ppm).

The precursor solution for 3D MAPbI_3_ perovskite is prepared by dissolving MAI and PbI_2_ with a molar ratio of 1:1.2 in a mixed solvent of 0.8 mL dimethylformamide (DMF) and 0.2 mL dimethyl sulfoxide (DMSO). The solution is stirred overnight at room temperature using a magnetic stirrer to ensure complete dissolution of the precursors in the solvent.

The precursor solution is spin-coated onto the ITO substrate using a spin-coating machine. The process is carried out in two stages of rotational speed. First, it spins at 2000 rpm for 10 s, and then the speed is switched to 6000 rpm and continues spinning for 30 s. The lower speed helps improve the dispersion of the solution and enables it to be uniformly coated on the substrate. The higher speed allows the coating to rapidly spin off, facilitating solvent evaporation and the formation of a denser and smoother film structure. Additionally, at the 20-s mark during the spinning process, 220 µL of the anti-solvent chlorobenzene is dripped to induce rapid crystallization of the perovskite. After spin-coating, the substrate is placed on a 100 °C hotplate for 30 min to evaporate any residual organic solvent and enhance the crystallinity of the film.

For the 2D perovskite with the chemical formula (BA)_2_Ma_n−1_Pb_n_I_3n+1_, different *n* values are achieved by controlling the volume molar concentration ratios of the precursors BAI, MAI, and PbI_2_. The detailed ratio relationships are listed in Table 1. The subsequent processing steps are the same as for the 3D perovskite, with the precursor dissolved in a mixed solvent of 0.8 mL DMF and 0.2 mL DMSO. A 100 µL volume of the precursor solution is spin-coated onto the ITO substrate and annealed at 100 °C for 30 min. The crystalline structure was examined by grazing incidence X-ray diffraction (GIXRD, Bruker D8 DISCOVER) with Cu-Kα (λ = 0.15406 nm) radiation. The X-ray incidence angle was set to 1°. The diffraction angle (2θ) was scanned from 2° to 30°.

## 3. Results and Discussion

Figure S4 depicts the temperature distribution maps of 3D perovskite with various sizes after completing molecular dynamics simulations. The temperature gradients between the hot and cold regions are calculated from these distribution maps, and the energy flow between the hot and cold regions is simultaneously recorded to calculate the heat flux. Each black point in the figure represents the average temperature of the thin layer in that region (1.3 nm). The simulated sizes range from 38.3 nm to 153.2 nm. It is evident that the positions and temperatures of the hot and cold regions align with the initially set conditions, and the temperature distribution between the hot and cold regions exhibits a highly linear pattern, contributing to the enhanced accuracy of subsequent thermal conductivity calculations.

Figure S5 presents the thermal conductivity calculated for three-dimensional calcium titanate based on the selected temperature range from Figure S4. Due to the highly linear relationship of temperature, the size of the selected range between 350 K and 250 K does not significantly impact the thermal conductivity. On the other hand, when the selected range is too small (with a large cutoff number), a noticeable deviation in the calculated thermal conductivity occurs. This is because a limited number of sampling points lead to a departure of the temperature gradient in that range from the overall trend.

Detailed data related to thermal conductivity calculations are summarized in Table 2, including heat flux ($\vec{q}$), temperature gradient (*dT*/*dx*), and thermal conductivity (*k*) calculated through Fourier’s law of heat conduction. It can be observed that as the simulation size increases, the thermal conductivity exhibits an approximately linear increasing trend. This phenomenon can be attributed to finite size effects. As the simulation size increases, the maximum wavelength of phonons allowed in the material also increases, indicating that more phonon modes can be excited, leading to an overall increase in thermal conductivity.

The initial part of the 3D perovskite involves 10 million steps of NPT simulation. To reduce computational time and costs, the NPT steps for the two-dimensional calcium titanate are reduced to 5 million steps while keeping the remaining process steps constant. Figure S6 compiles the changes in potential energy with the number of steps for different values of *n* in 2D perovskite with the same size (38.3 nm). The red line represents the average potential energy over the last 1 million steps, demonstrating that even with the reduction in NPT steps to 5 million, it is sufficient time for the system’s potential energy to reach a steady state and oscillate around a certain value.

Figure S8 illustrates the thermal conductivity calculated for 2D perovskite based on the selected temperature range from Figure S7. Due to the relatively scattered temperature distribution in Figure S7f, the thermal conductivity calculated in Figure S8f is influenced by the range chosen between the hot and cold regions. This significantly impacts the accuracy of the simulation results. Therefore, for the thermal conductivity calculation model of 2D perovskite, only the first five data points for sizes ranging from 38.3 nm to 130.2 nm are considered.

**Figure 3:**
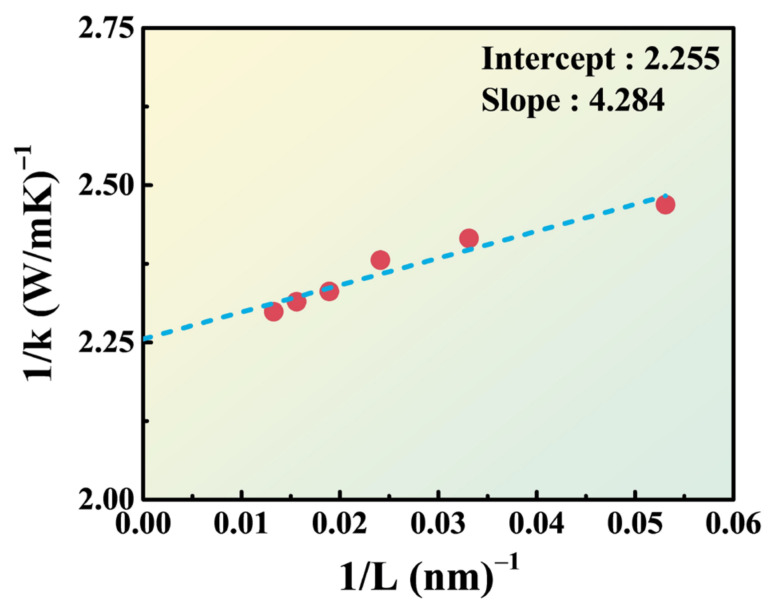
The calculated and fitted results of the thermal conductivity extrapolation of 3D perovskite.

Figure 3 represents the computed thermal conductivity of 3D perovskite (MAPbI_3_) as L → ∞ using extrapolation. The *x*-axis corresponds to the reciprocal of length, while the *y*-axis represents the reciprocal of the calculated thermal conductivity. By fitting these data to a linear equation, the thermal conductivity at L → ∞ can be determined. The thermal conductivity of MAPbI_3_ computed is approximately 1/2.255 ≈ 0.443 W/m-K. Note that the thermal conductivity of MAPbI_3_ was experimentally measured using frequency domain thermos-reflectance as 0.34 ± 0.08 W/m-K [33]. It is worth noting that metal halide perovskite materials generally exhibit low thermal conductivities, typically below 1 W/m-K. The slight discrepancy between the simulated and measured values may be attributed to the ideal defect-free perovskite structure assumed in the simulations, whereas real materials often possess some lattice defects that can affect phonon transport. This can lead to slightly higher simulated values compared to the experimental measurements.

Due to the orientation dependence based on the crystal structure arrangement, 2D perovskite can be classified into different orientations: parallel or perpendicular. Therefore, the model construction and placement of the hot and cold regions are designed accordingly, as displayed in Figures S9 and S10. For the parallel orientation, only the unit cell along the z direction was replicated based on different lengths of the unit cell. The hot and cold regions were positioned along the z direction, allowing the heat flow to propagate along the z direction. In contrast, for perpendicular orientation, the unit cell along the x direction was replicated based on different lengths of the unit cell. The hot and cold regions are positioned along the x direction, enabling the heat flow to propagate along the x direction.

**Figure 4:**
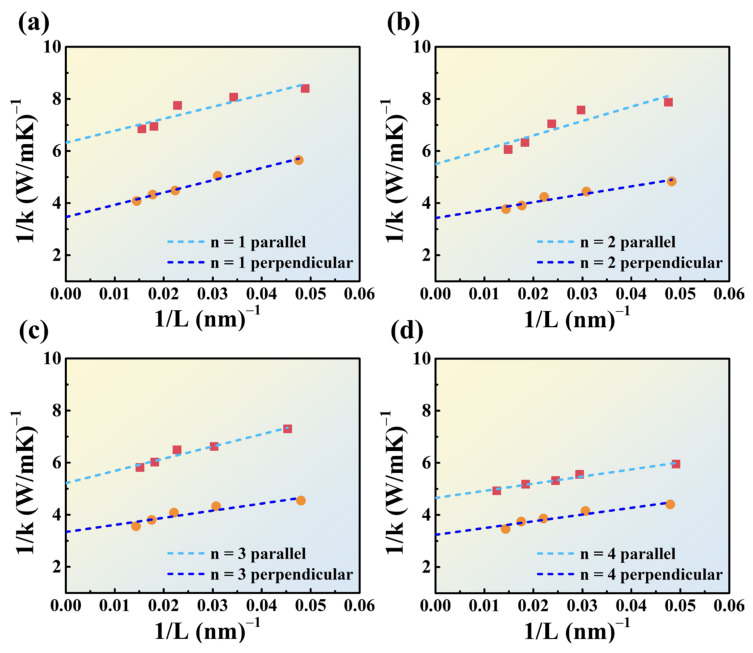
The calculated and fitted results of the thermal conductivity extrapolation of 2D perovskites in the parallel orientation (red squares) and perpendicular orientation (orange circles), (**a**) *n* = 1, (**b**) *n* = 2, (**c**) *n* = 3, and (**d**) *n* = 4.

**Figure 5:**
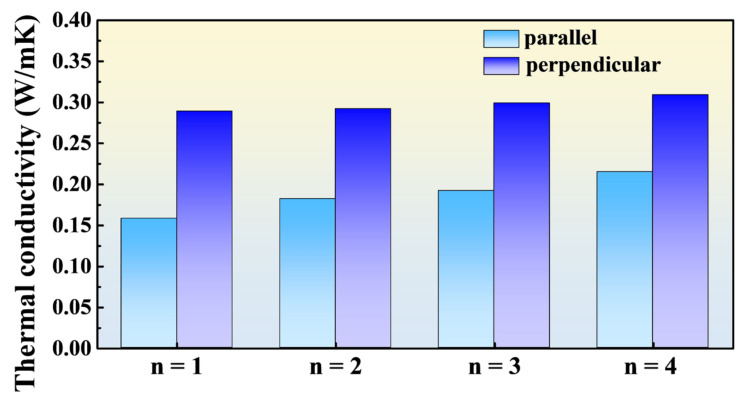
Bar chart of 2D perovskite thermal conductivity extrapolation.

The extracted thermal conductivities are displayed in Figure 4 and Figure 5 and summarized in Table 3, indicating that regardless of the 2D perovskite orientation (parallel or perpendicular), the thermal conductivity increases with an increase in the value of *n*. This confirms that an increase in the number of inorganic octahedral layers in 2D perovskite promotes phonon transport and enhances thermal conductivity.

For 3D perovskite, its overall structure consists primarily of inorganic octahedral structures, resulting in a noticeably higher thermal conductivity of 0.443 W/m-K. It also confirms that the presence of organic long-chain cations in 2D perovskite effectively reduces the thermal conductivity. The literature reports the measured thermal conductivity using time-domain thermos-reflectance, showing that for *n* = 1 (BA)_2_PbI_4_, it is 0.18 ± 0.04 W/mK, which is lower than the thermal conductivity of 3D perovskite [34].

When the 2D perovskite is arranged in the parallel orientation, the thermal conductivity is consistently lower than that in the perpendicular orientation. This suggests that when the inorganic octahedral structures in the perovskite are fully encapsulated by the organic long-chain spacers, the phonon transport is effectively impeded. This leads to an orientation-dependent thermal conductivity for 2D perovskite of identical *n* values. Interestingly, in the parallel orientation, the thermal conductivities are more sensitive to the *n* values relative to those of the perpendicular orientation. This suggests that if the number of inorganic octahedral layers (*n* value) is sufficiently large, phonons can overcome the obstacles imposed by the organic long-chain spacers above/below to transfer thermal energy.

**Figure 6:**
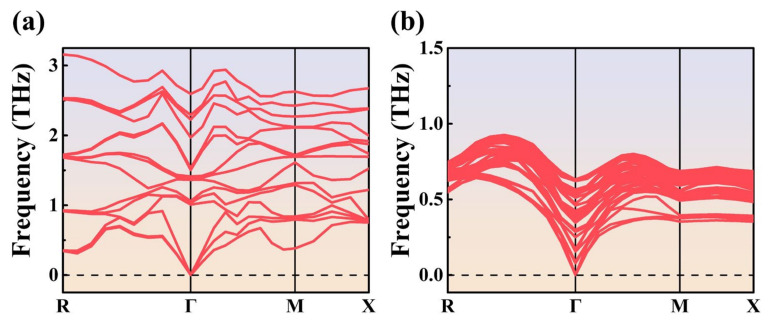
Phonon dispersion of (**a**) 3D perovskite (**b**) 2D perovskite at 300 K. The horizontal axis is the wave number in units of nm^−1^, and R, Γ, M, and X represent four high-symmetry points in the first Brillouin zone, indicating different wave vector positions. The vertical axis shows the phonon frequency in units of THz. The phonon group velocity can be calculated by determining the slope near the Γ point.

The inferior thermal conductivity of 2D perovskites relative to their 3D counterparts can be validated by computing the phonon group velocities from phonon dispersion curves. Figure 6 displays the phonon dispersion curves of 3D (Figure 6a) and *n* = 2 2D (Figure 6b) perovskites. The phonon group velocities can be computed by extracting the slope near the Γ point. The calculation results indicate that the phonon group velocity of MAPbI_3_ at low frequencies is 2.42 km/h, while that of the (BA)_2_MAPb_2_I_7_ is 1.95 km/h. Hence, the phonon group velocity in 3D perovskite is approximately 1.2 times faster than that of 2D perovskite. This further confirms that the organic long-chain cations in 2D perovskite have the characteristic of reducing the phonon group velocity. This material property demonstrates that 2D perovskite has a lower thermal conductivity compared to 3D perovskite.

**Figure 7:**
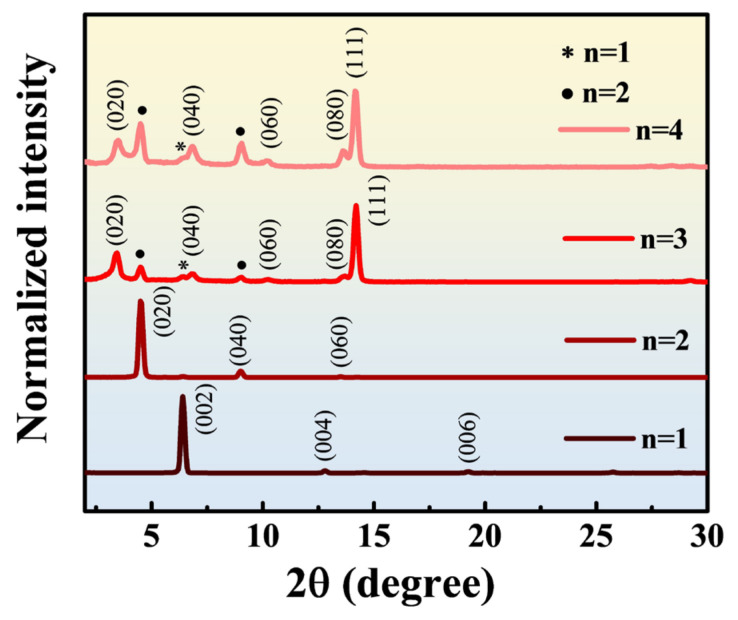
The GIXRD patterns of 2D perovskite with *n* = 1 to 4.

In Figure 7, a comparison of the grazing incidence X-ray diffraction (GIXRD) patterns for single-layer 2D perovskite is shown with an X-ray incidence angle set at 1°. For *n* = 1, the diffraction peaks appear sequentially at 6.4° (002), 12.8° (004), and 19.2° (006). For *n* = 2, the diffraction peaks appear sequentially at 4.5° (020), 9.0° (040), and 13.5° (060). In both cases, the materials exhibit higher characteristic peak intensities at low angles.

For *n* = 3 and 4, the GIXRD patterns show similar results. The diffraction peaks appear sequentially at 3.5° (020), 12.8° (040), 10.3° (060), 13.7° (080), and 14.2° (111). The diffraction peak intensity is more significant for the (111) crystal plane. Additionally, for both materials, a diffraction peak signal of *n* = 1 (002) appears at 6.4°, and signals of *n* = 2 (020) and (040) appear at 4.5° and 9.0°, respectively. This suggests that when preparing 2D perovskite with higher *n* values (*n* = 3 and 4), lower *n*-value (*n* = 1 and 2) 2D perovskite is simultaneously generated on the surface of the thin film. Therefore, the film composition exhibits a mixture and distribution of multiple *n* values.

Figure S11 shows the PL spectra. In the case of 2D perovskites with *n* values of 1 and 2 or 3D perovskites, the PL exhibit a single peak at varying wavelengths, indicative of differences in bandgap. As the *n* value increases, a redshift in the peak position signifies a reduction in the bandgap energy. Conversely, when *n* equals 3 or 4, the spectra exhibit multiple peaks, suggesting the coexistence of various *n* values within the material’s structure. Additionally, a supplementary spectral signal emerges at around 750 nm, slightly below the wavelength of the three-dimensional perovskite peak at 775 nm. This signal implies the formation of two-dimensional perovskite phases with larger *n* values (>5).

The power factor, S^2^σ, is used to calculate the thermoelectric figure of merit. First, the Seebeck coefficient was investigated for different *n* values of 2D perovskite and 3D perovskite. The Seebeck coefficient measurements were conducted at room temperature, with 4 cm between the hot and cold ends and a temperature difference controlled around 50 K.

**Figure 8:**
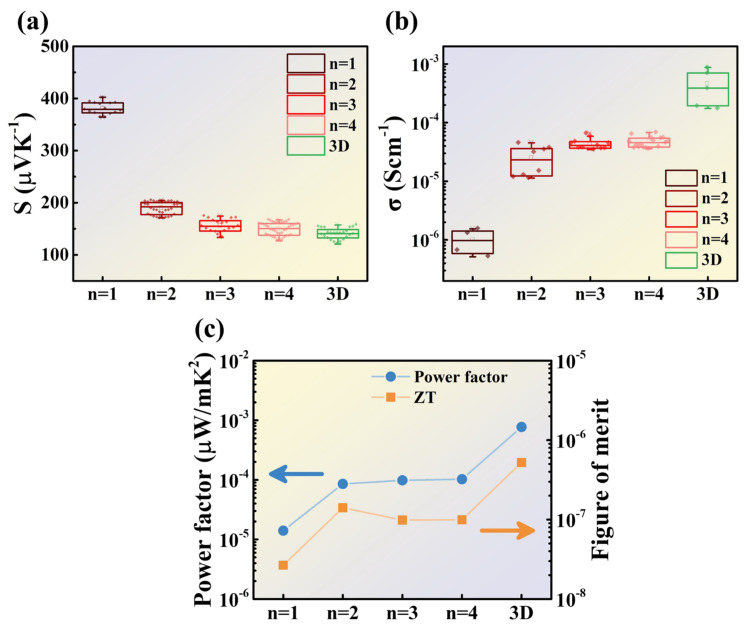
(**a**) Electrical conductivity (**b**) Seebeck coefficient (**c**) power factor and ZT value.

The Seebeck coefficient measurements are shown in Figure 8a. It is evident that the overall trend is that the Seebeck coefficient decreases as the *n* value increases. When *n* = 3 or 4, the Seebeck coefficient approaches that of 3D perovskite. Only for *n* = 1 is the Seebeck coefficient significantly higher than that of other perovskite materials. Comparing the median values of each dataset, the Seebeck coefficient for *n* = 1 is approximately 2.7 times higher than that of 3D perovskite.

Two-dimensional perovskite contains long-chain organic cations, which can form natural quantum well structures within the material to enhance the density of electronic states. According to the literature, it has been theoretically proven that increasing the density of states can effectively improve the Seebeck coefficient. In the same material, low-dimensional structures such as 2D quantum wells and 1D quantum wires exhibit higher Seebeck coefficients compared to bulk materials [35].

Subsequently, the Hall effect analyzer was used to measure the electrical conductivity of the perovskite. Figure 8b shows the overall trend, which is opposite to that of the Seebeck coefficient. The electrical conductivity increases as the *n* value increases. Comparing the median values of each dataset, the electrical conductivity of 3D perovskite is higher than that of *n* = 2, 3, and 4 by approximately one order of magnitude. Additionally, the electrical conductivity of *n* = 2, 3, and 4 in 2D perovskite is also approximately one order of magnitude higher than that of *n* = 1.

Table 4 summarizes the relevant data obtained from measuring the electrical conductivity. The carrier concentration for all types of perovskites falls within the range of 10^12^ cm^−3^. As the *n* value increases, the carrier concentration slightly increases. However, the carrier mobility shows a significant improvement with increasing *n* values. The difference in carrier mobility between *n* = 1 and 3D perovskite is two orders of magnitude, confirming that the electrical conductivity of perovskite is primarily influenced by carrier mobility. This also suggests that the electron transport in perovskite relies on the Pb-I inorganic octahedral structure, while the presence of organic long-chain cations hinders carrier mobility and thus lowers the electrical conductivity. The highest proportion of organic long-chain cations in *n* = 1 perovskite leads to the lowest electrical conductivity, only 9.76 × 10^−7^ s/cm. On the other hand, 3D perovskite, which does not contain organic long-chain cations, exhibits higher electrical conductivity than any type of 2D perovskite.

The calculated power factor and thermoelectric figure of merit values, using the measured Seebeck coefficient and electrical conductivity, are summarized in Figure 8c and Table 5. The power factor of 2D perovskite is overall more than one order of magnitude lower than that of 3D perovskite. Specifically, the power factor of *n* = 1 in 2D perovskite is significantly lower than that of other *n* values. The power factors calculated for *n* = 2, 3, and 4 show a slight increasing trend. Despite the advantage of a higher Seebeck coefficient in 2D perovskite, the electrical conductivity being more than one order of magnitude lower than that of 3D perovskite leads to a similar trend in the power factor.

In Figure 8c, the thermoelectric figure of merit is calculated by substituting the power factor and the previously simulated thermal conductivity into Equation (1). Based on the previous analysis of low-angle diffraction crystal orientations, the thermal conductivities used for *n* = 1 and 2 are from the parallel orientation, while for *n* = 3 and 4, the thermal conductivities from the perpendicular orientation are used.

The calculation results show that *n* = 2 has the highest thermoelectric figure of merit in 2D perovskite. Although there is not a significant difference in the power factor among *n* = 2, 3, and 4, the material characteristic of *n* = 2, with its parallel orientation and lower thermal conductivity, allows it to stand out in terms of thermoelectric figure of merit in 2D perovskite. On the other hand, despite the advantage of a higher Seebeck coefficient and lower thermal conductivity in 2D perovskite compared to 3D perovskite, the disadvantage of having an electrical conductivity difference of more than one order of magnitude results in an overall superior thermoelectric figure of merit in 3D perovskite.

Based on the combined thermal and electrical data of perovskite, it can be inferred that the bilayer structure of 2D and 3D perovskite can leverage the respective advantages of both materials to enhance the overall thermoelectric figure of merit. The underlying 3D perovskite provides higher carrier mobility, improving electrical conductivity. Meanwhile, the upper layer of 2D perovskite, particularly *n* = 2 with a parallel orientation, effectively reduces thermal conductivity. Additionally, at the interface between the2D and 3D perovskite layers, there can be an additional interface of thermal resistance that disrupts phonon transport. However, the impact of the upper layer of 2D perovskite on the overall electrical conductivity should also be investigated. Ideally, the positive benefits of reduced thermal conductivity and an increased Seebeck coefficient should outweigh the negative impact of reduced electrical conductivity.

## 4. Conclusions

The simulation results of this study showed that 2D perovskites with *n* = 1–4 have lower thermal conductivity than 3D perovskites, regardless of their arrangement orientation. It indicates that the long organic chain cations in 2D perovskites effectively impede phonon transfer. This phenomenon is confirmed by calculating the phonon group velocities using the phonon dispersion relation, where the phonon group velocity of 2D perovskites is 1.95 km/h and that of 3D perovskites is 2.42 km/h, with a difference of approximately 20%. The arrangement orientation in 2D perovskites also affects the calculated thermal conductivity, as 2D perovskites with orientations from *n* = 1 to *n* = 4 in the horizontal direction have lower thermal conductivity than those in the vertical direction. This indicates that when perovskites are used as thermoelectric materials, their inorganic octahedral structures serve as pathways for both electron and phonon transport. It is also observed that the difference in thermal conductivity between orientations *n* = 1 to *n* = 4 is more significant in the horizontal direction than in the vertical direction. This suggests that as long as the number of inorganic octahedral layers (*n* value) is sufficiently large, phonons can still overcome the hindrance of the long organic chain cations at the top and bottom ends to transmit heat, thereby significantly increasing the thermal conductivity.

In terms of thermoelectric performance, we found that 2D perovskites have the advantage of a high Seebeck coefficient and low thermal conductivity compared to 3D perovskites. However, due to the much higher electrical conductivity of 3D perovskites, with a difference of approximately one order of magnitude or more, the overall ZT is still higher for 3D perovskites. Among the 2D perovskites, those with *n* = 2 exhibit the highest ZT. It is suggested that future research could focus on designing bilayer structures with a combination of 2D and 3D perovskites to leverage their respective advantages and enhance the thermoelectric ZT. The experimentally measured ZT of perovskites shows a significant discrepancy compared to the relevant simulation literature. This is mainly because the simulation literature generally overestimates the electrical conductivity of perovskites. To achieve the maximum thermoelectric ZT, the carrier concentration in perovskites needs to be increased to 10^19^ cm^−3^, while the carrier concentration measured by Hall effect analysis for most perovskites are approximately 10^12^ cm^−3^, leading to a very low ZT. Therefore, this study also suggests improving the electrical conductivity of perovskites by increasing the carrier concentration to enhance the overall thermoelectric ZT.

## Figures and Tables

**Table 1:** Stoichiometric ratio of the different *n* value 2D perovskite.

Sample	BAI	MAI	PbI_2_
*n* = 1	2	0	1
*n* = 2	2	1	2
*n* = 3	2	2	3
*n* = 4	2	3	4

**Table 2:** Simulation of thermal conductivity in 3D perovskite with varying length.

Length (nm)	$\vec{q}$ (W/m^2^)	*−dT/dx* (K/m)	k (W/mK)
38.3	1.89 × 10^9^	4.66 × 10^9^	0.405
61.3	1.20 × 10^9^	2.91 × 10^9^	0.414
84.3	9.11 × 10^8^	2.17 × 10^9^	0.420
107.2	7.34 × 10^8^	1.71 × 10^9^	0.429
130.2	6.35 × 10^8^	1.47 × 10^9^	0.432
153.2	5.26 × 10^8^	1.21 × 10^9^	0.435

**Table 3:** Extrapolated values of thermal conductivity for 2D perovskite.

Sample	Parallel (W/mK)	Vertical (W/mK)
*n* = 1	0.158	0.289
*n* = 2	0.182	0.292
*n* = 3	0.192	0.299
*n* = 4	0.215	0.309

**Table 4:** Carrier concentration, mobility, and electrical conductivity of perovskite.

Sample	*n* (cm^−3^)	μ (cm^2^/V)	σ (s/cm)
*n* = 1	2.32 × 10^12^	2.63 × 10^0^	9.76 × 10^−7^
*n* = 2	3.58 × 10^12^	4.05 × 10^1^	2.32 × 10^−5^
*n* = 3	3.86 × 10^12^	6.62 × 10^1^	4.09 × 10^−5^
*n* = 4	4.16 × 10^12^	6.83 × 10^1^	4.55 × 10^−5^
3D	8.02 × 10^12^	3.02 × 10^2^	3.88 × 10^−4^

**Table 5:** Thermoelectric parameters of perovskite.

Sample	S (µV/K)	σ (s/cm)	k (W/mK)	ZT
*n* = 1	3.79 × 10^2^	9.76 × 10^−7^	0.158	2.66 × 10^−8^
*n* = 2	1.92 × 10^2^	2.32 × 10^−5^	0.182	1.41 × 10^−7^
*n* = 3	1.55 × 10^2^	4.09 × 10^−5^	0.299	9.86 × 10^−8^
*n* = 4	1.50 × 10^2^	4.55 × 10^−5^	0.309	9.94 × 10^−8^
3D	1.41 × 10^2^	3.88 × 10^−4^	0.443	5.22 × 10^−7^

## Data Availability

Data are contained within the article and Supplementary Materials.

## Supplementary Materials

The following supporting information can be downloaded at: https://www.mdpi.com/article/10.3390/nano14050446/s1, Figure S1: The variation of potential energy and temperature with the number of steps, (a,b) for the 3D perovskite, and (c,d) for the 2D perovskite. Figure S2: Simulate the temperature distribution and sampling range for (a,c,e) of the 3D perovskite, and for (b,d,f) of the cutoff results for thermal conductivity with the temperature sampling range. Figure S3: Simulate the temperature distribution and sampling range for (a,c,e) of the 2D perovskite, and for (b,d,f) of the cutoff results for thermal conductivity with the temperature sampling range. Figure S4: The temperature distribution maps of 3D perovskite with various sizes after completing molecular dynamics simulations. Figure S5: The relationship between the number of removed thin layers and thermal conductivity in 3D perovskite at different sizes. Figure S6: The potential energy variation with the number of steps for 2D perovskite at different *n* values, (a) *n* = 1, (b) *n* = 2, (c) *n* = 3, (d) *n* = 4. Figure S7: Temperature distribution of 2D perovskite at different lengths. Figure S8: The relationship between the number of removed thin layers and thermal conductivity in 2D perovskite at different sizes. Figure S9: 2D perovskite with (a) parallel (b) perpendicular orientation and heat flux direction. Figure S10: Diagram of 2D perovskite with (a) parallel (b) perpendicular orientation and heat flux direction. Figure S11: Photoluminescence analyses of (a–d) 2D and (e) 3D perovskite.
