# Supplementary material for: Comparative Study of the Orientation and Order Effects on the Thermoelectric Performance of 2D and 3D Perovskites
Source: Nanomaterials (Basel). 2024 Feb 28;14(5):446. doi: 10.3390/nano14050446 (PMC10935240; doi:10.3390/nano14050446)
Supplement: Supplementary file 1 [file nanomaterials-14-00446-s001.zip › nanomaterials-2887722-supplementary.pdf]

Supplementary Information

Article

Comparative study of the orientation and order effects on the thermoelectric performance of 2D and 3D perovskites

Yi-Hsiang Wang [1,†], Cheng-Hsien Yeh [1,†], I-Ta Hsieh [2], Po-Yu Yang [2], Yuan-Wen Hsiao [1], Hsuan-Ta Wu [3], Chun-Wei Pao [2*], and Chuan-Feng Shih [1,4,5*]

[1] Department of Electrical Engineering, National Cheng Kung University, Tainan 70101, Taiwan

[2] Research Center for Applied Sciences, Academia Sinica, Taipei 11529, Taiwan

[3] Department and Institute of Electrical Engineering, Minghsin University of Science and Technology, Hsinchu 30401, Taiwan

[4] Applied High Entropy Technology (AHET) Center, National Cheng Kung University, Tainan 70101, Taiwan

[5] Hierarchical Green-Energy Materials (Hi-GEM) Research Center, National Cheng Kung University, Tainan 70101, Taiwan

[*] Correspondence: cfshih@mail.ncku.edu.tw (C.F.S.); cwpao@gate.sinica.edu.tw (C.W.P.)

[†] These authors contributed equally to this work.

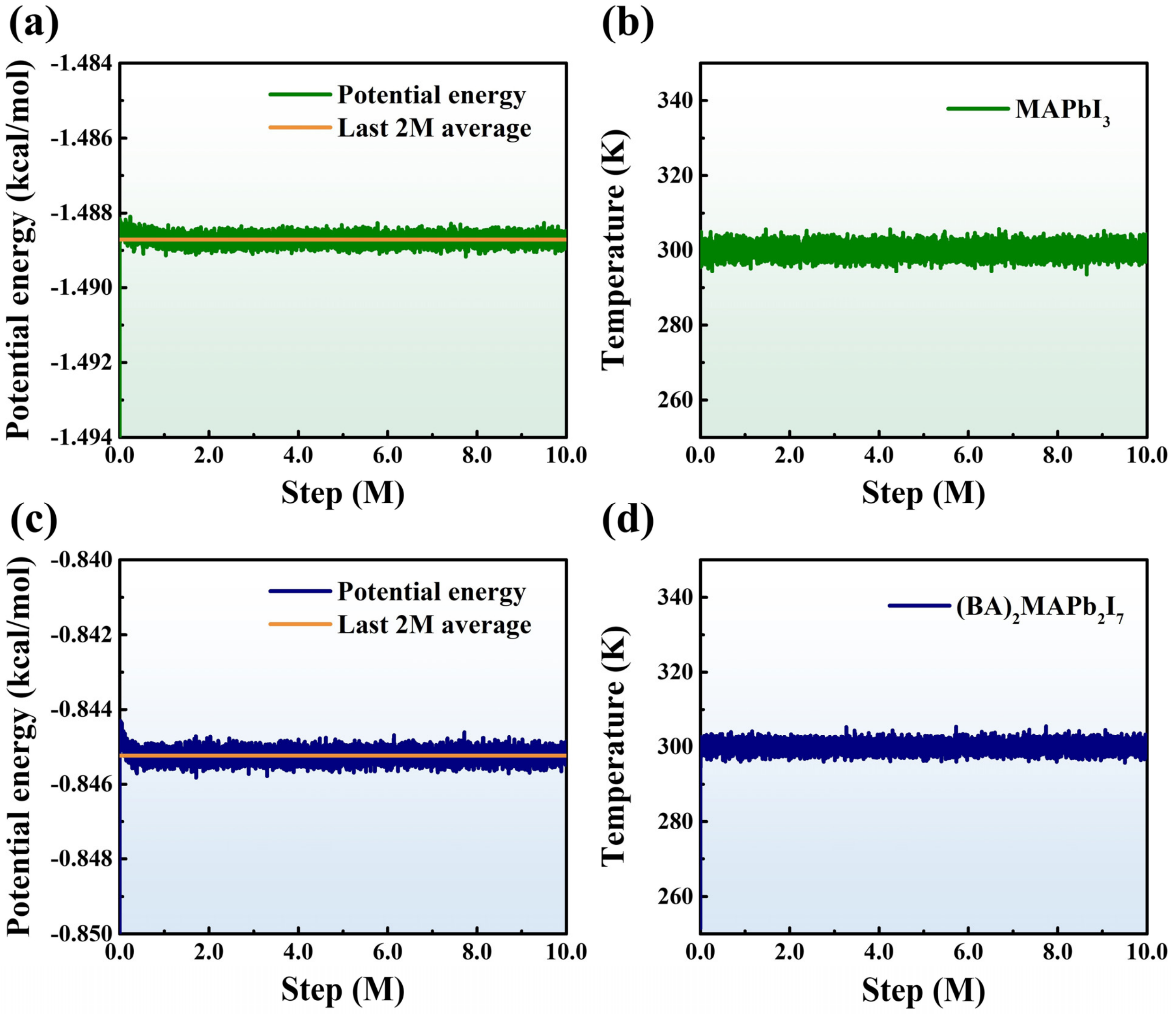

Figure S1. The variation of potential energy and temperature with the number of steps, (a)(b) for the 3D perovskite, and (c)(d) for the 2D perovskite.

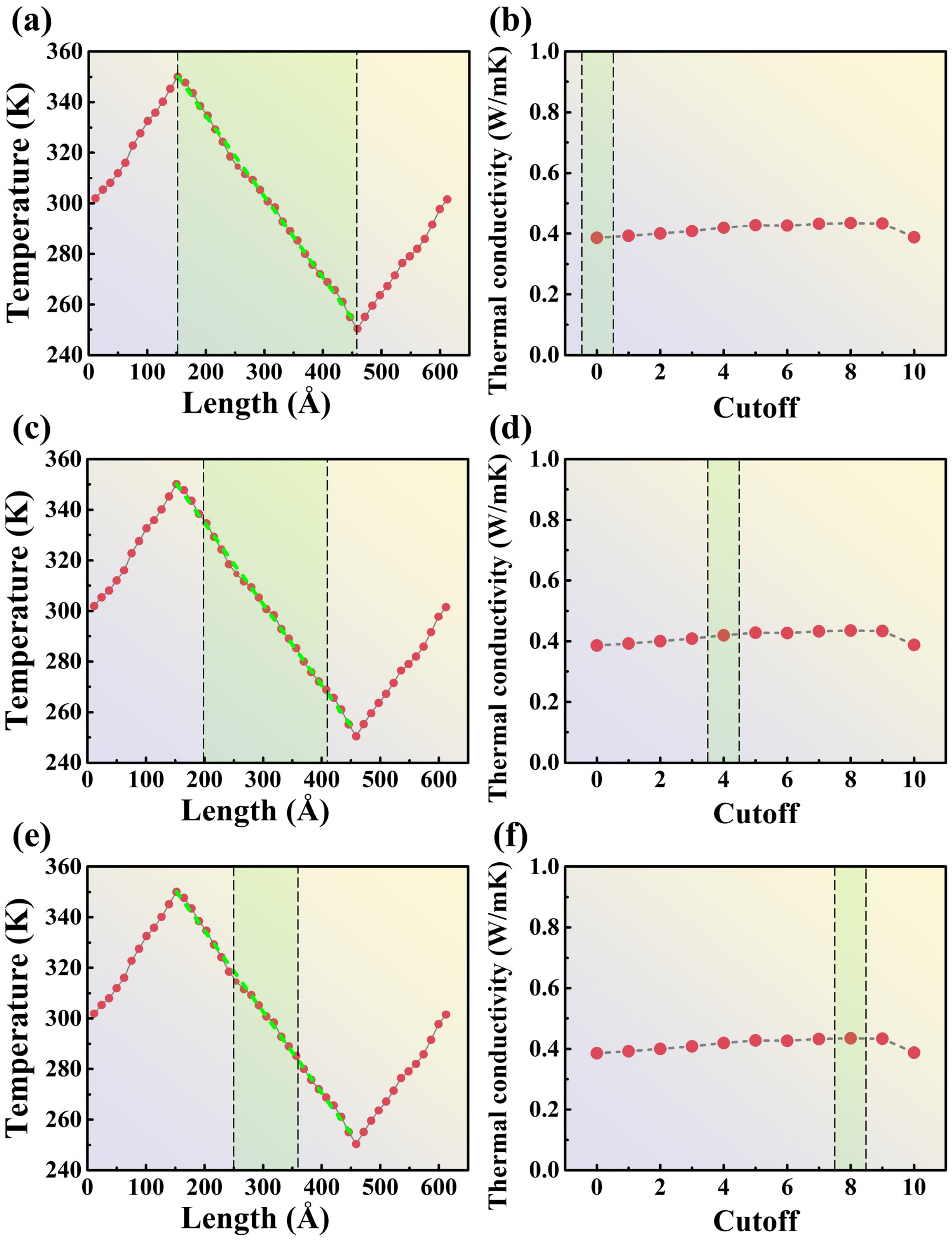

Figure S2. Simulate the temperature distribution and sampling range for (a)(c)(e) of the 3D perovskite, and for (b)(d)(f) of the cutoff results for thermal conductivity with the temperature sampling range.

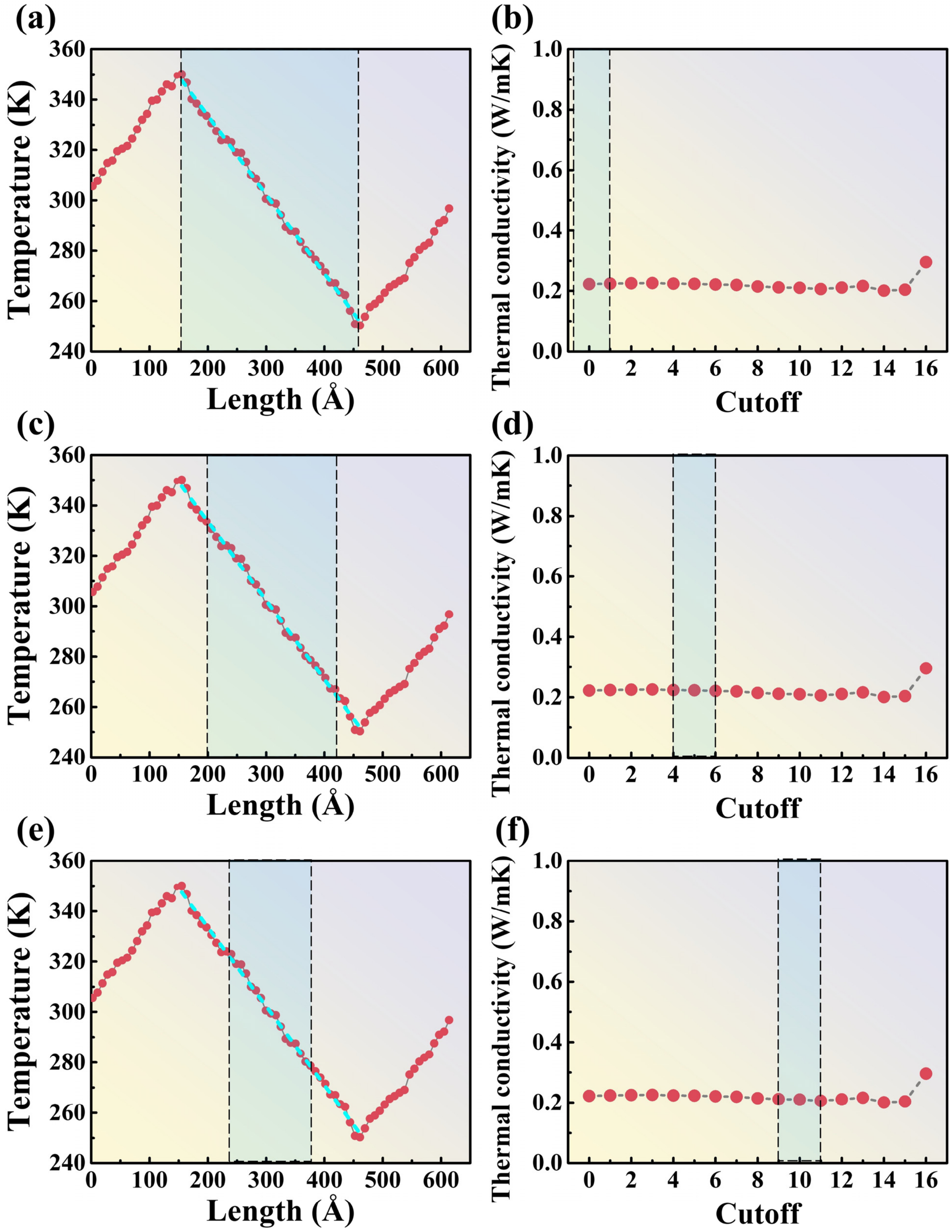

Figure S3. Simulate the temperature distribution and sampling range for (a)(c)(e) of the 2D perovskite, and for (b)(d)(f) of the cutoff results for thermal conductivity with the temperature sampling range.

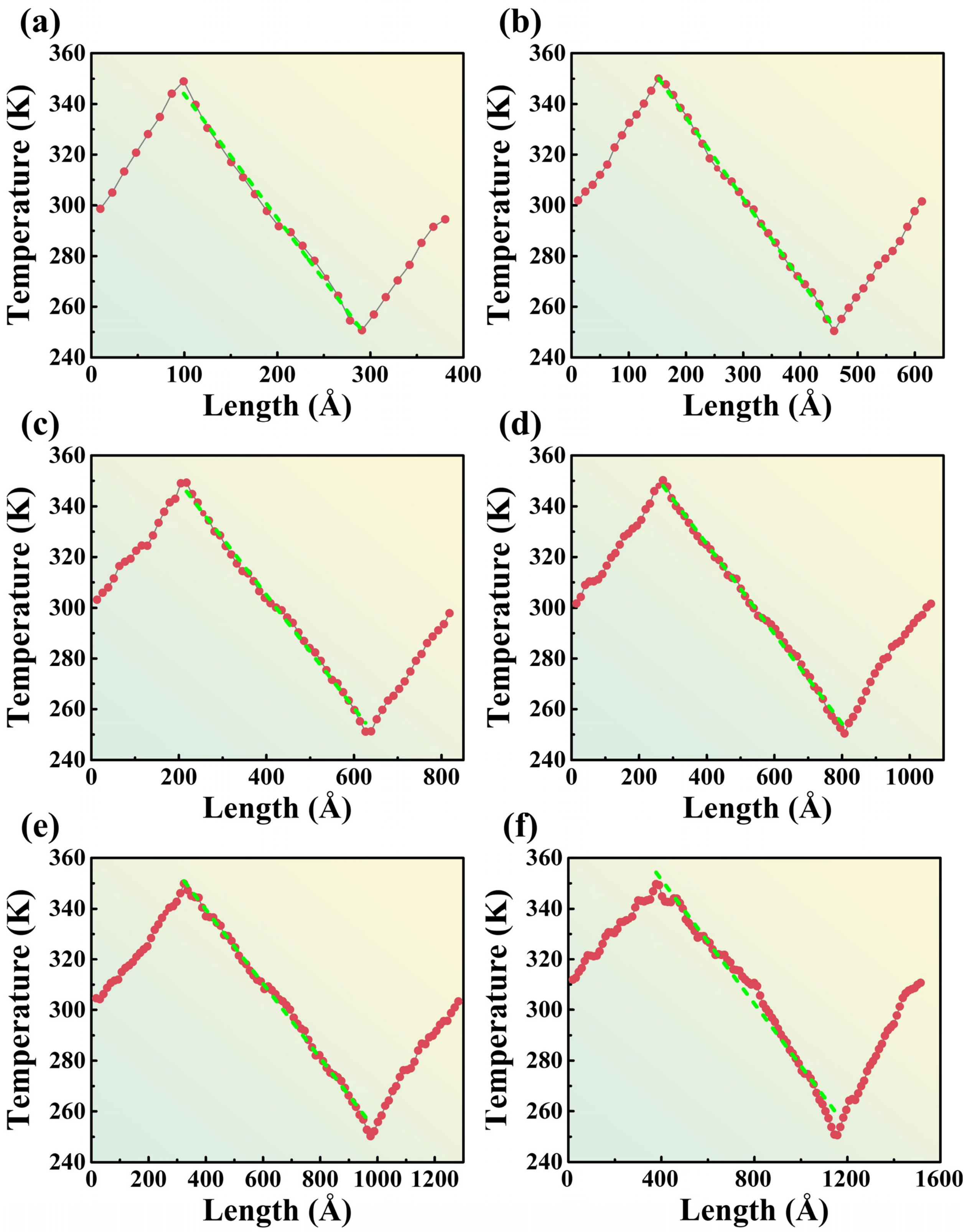

Figure S4. The temperature distribution maps of 3D perovskite with various sizes after completing molecular dynamics simulations.

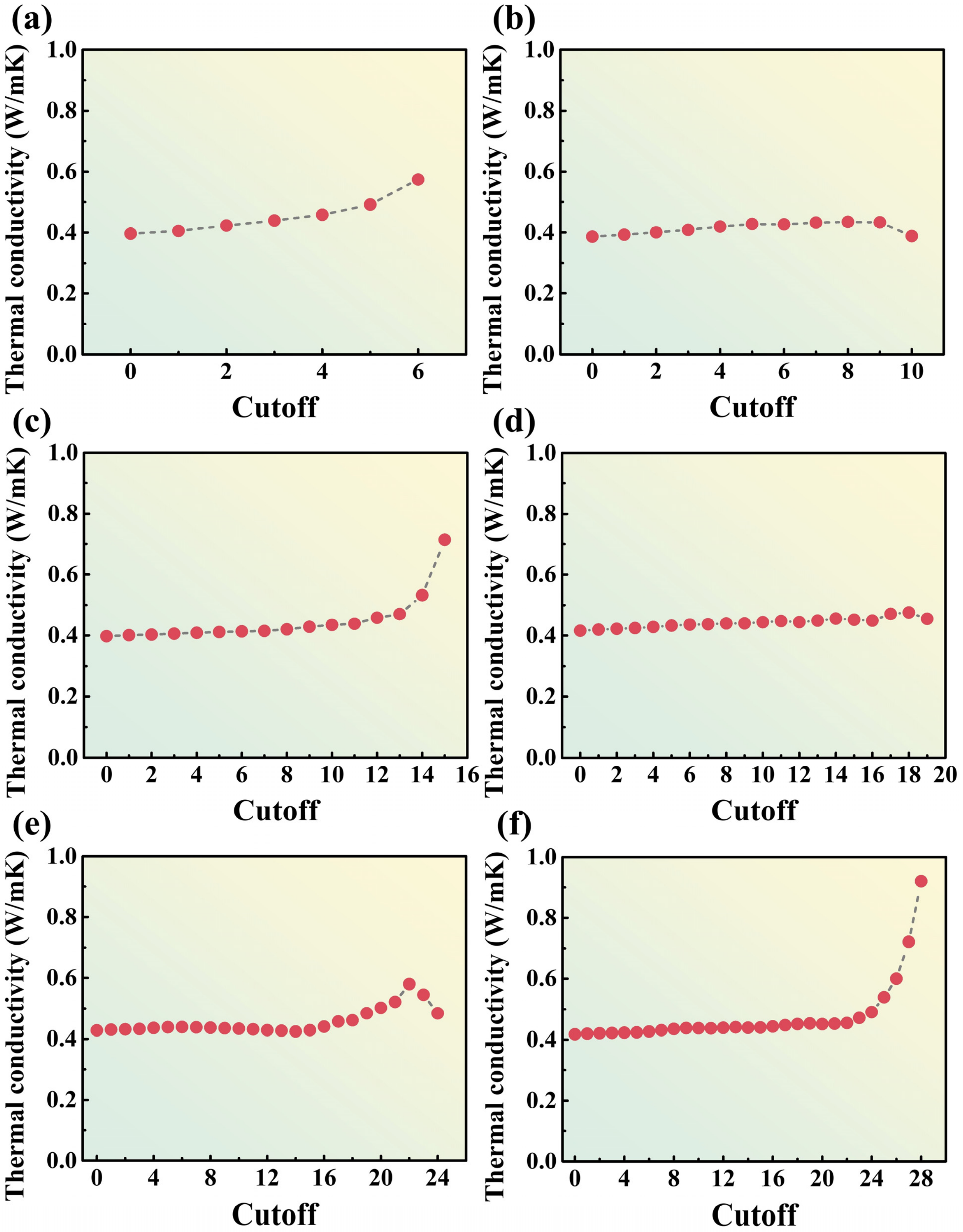

Figure S5. The relationship between the number of removed thin layers and thermal conductivity in 3D perovskite at different sizes.

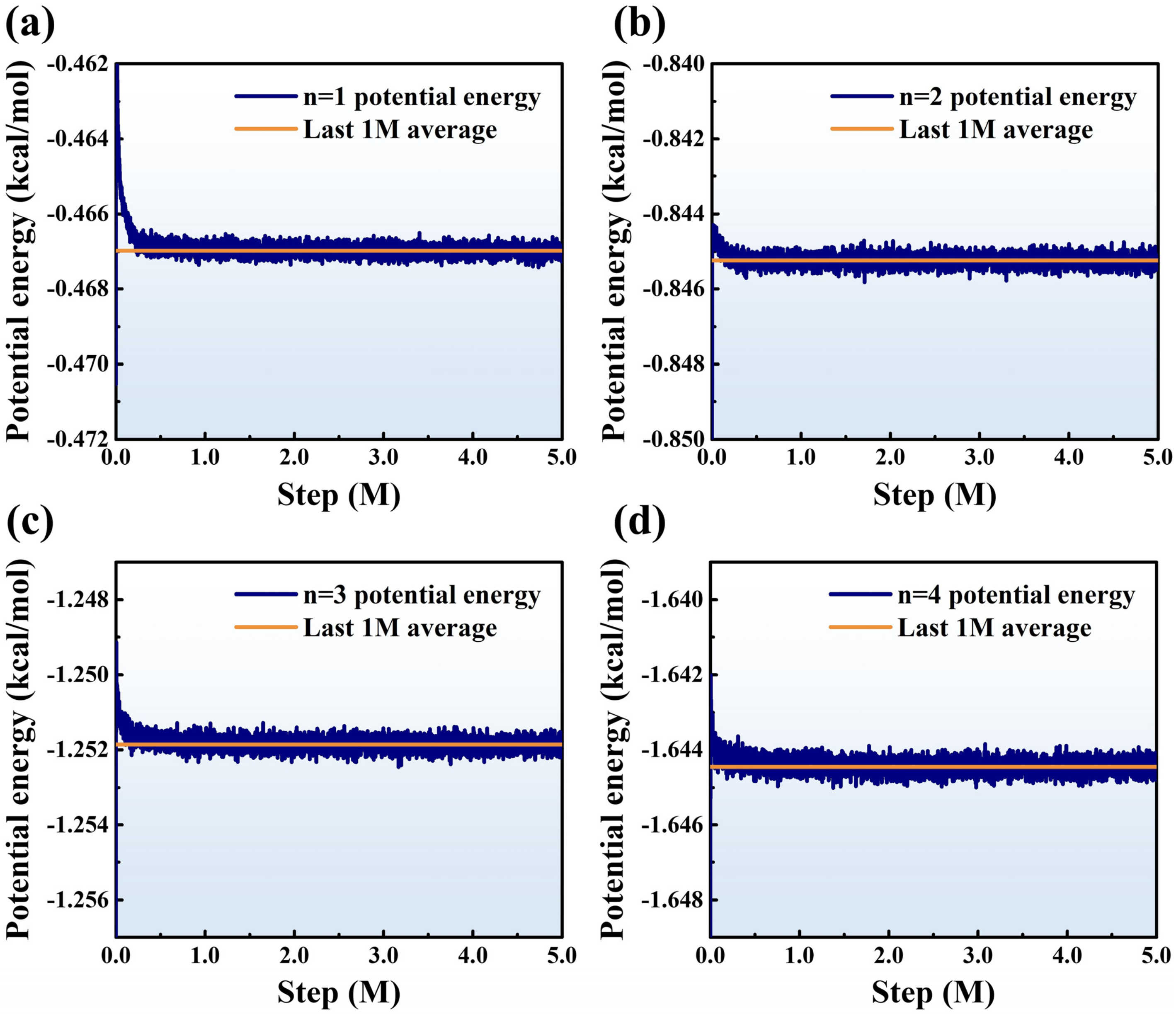

Figure S6. The potential energy variation with the number of steps for 2D perovskite at different n values, (a) n = 1, (b) n = 2, (c) n = 3, (d) n = 4.

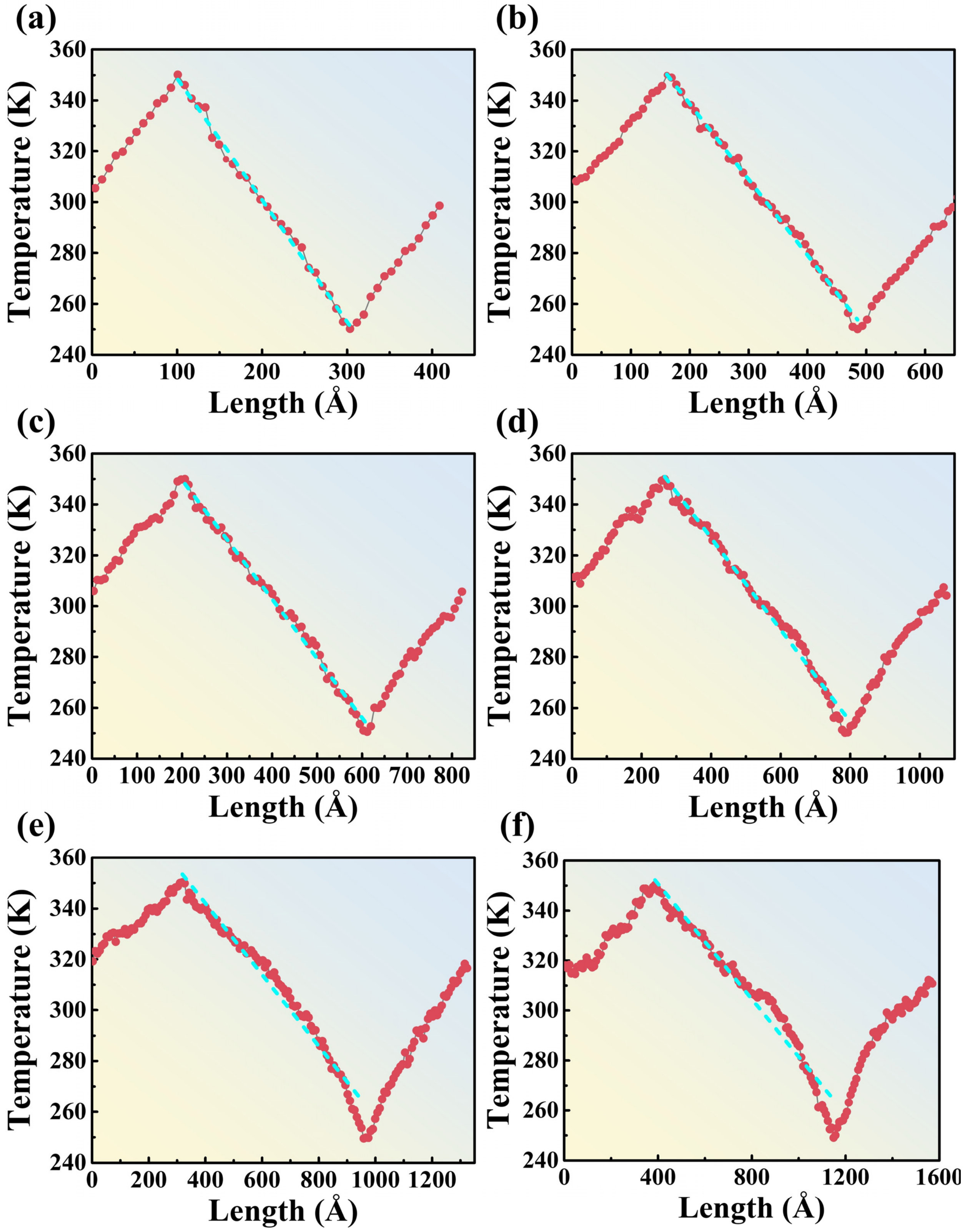

Figure S7. Temperature distribution of 2D perovskite at different lengths.

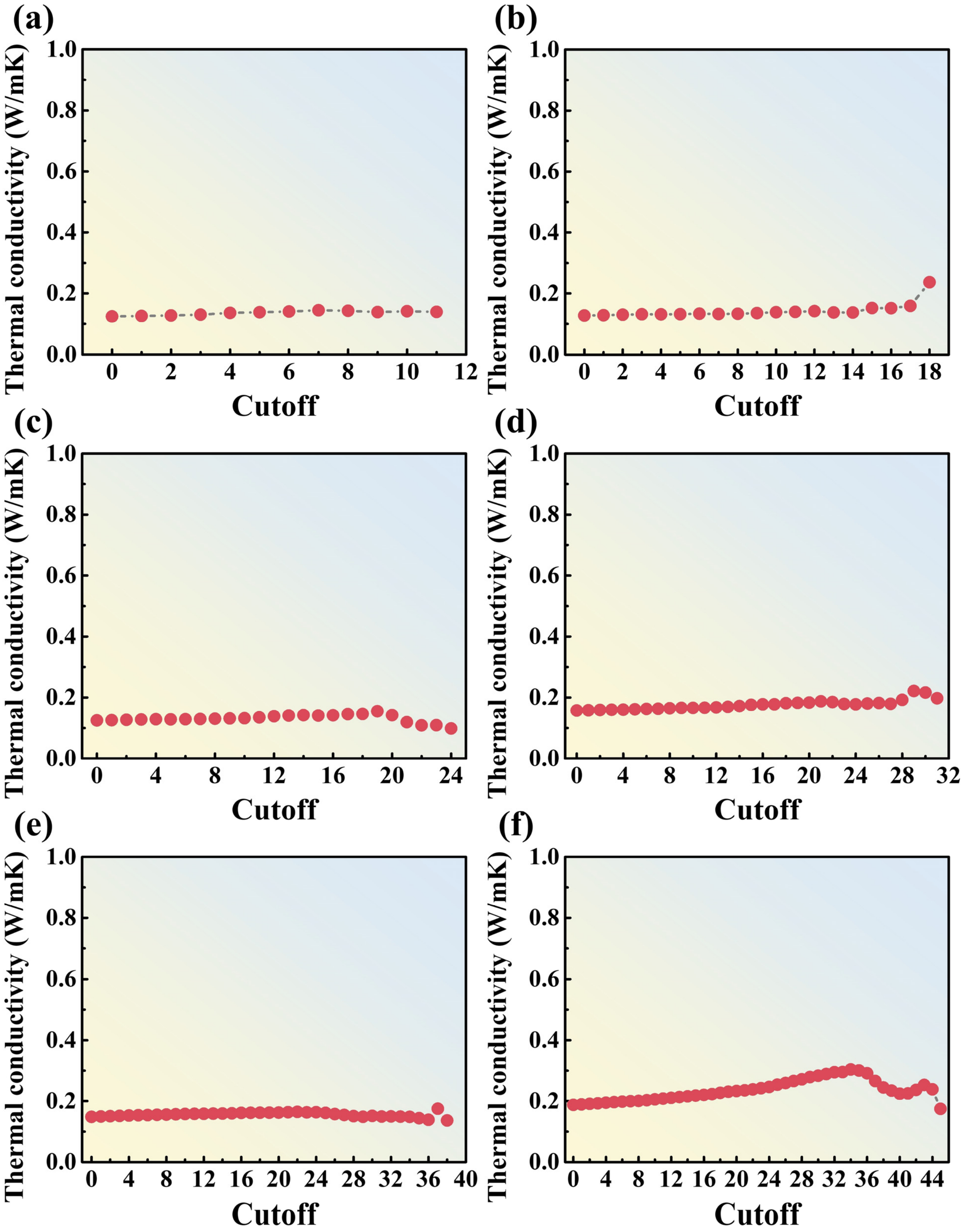

Figure S8. The relationship between the number of removed thin layers and thermal conductivity in 2D perovskite at different sizes.

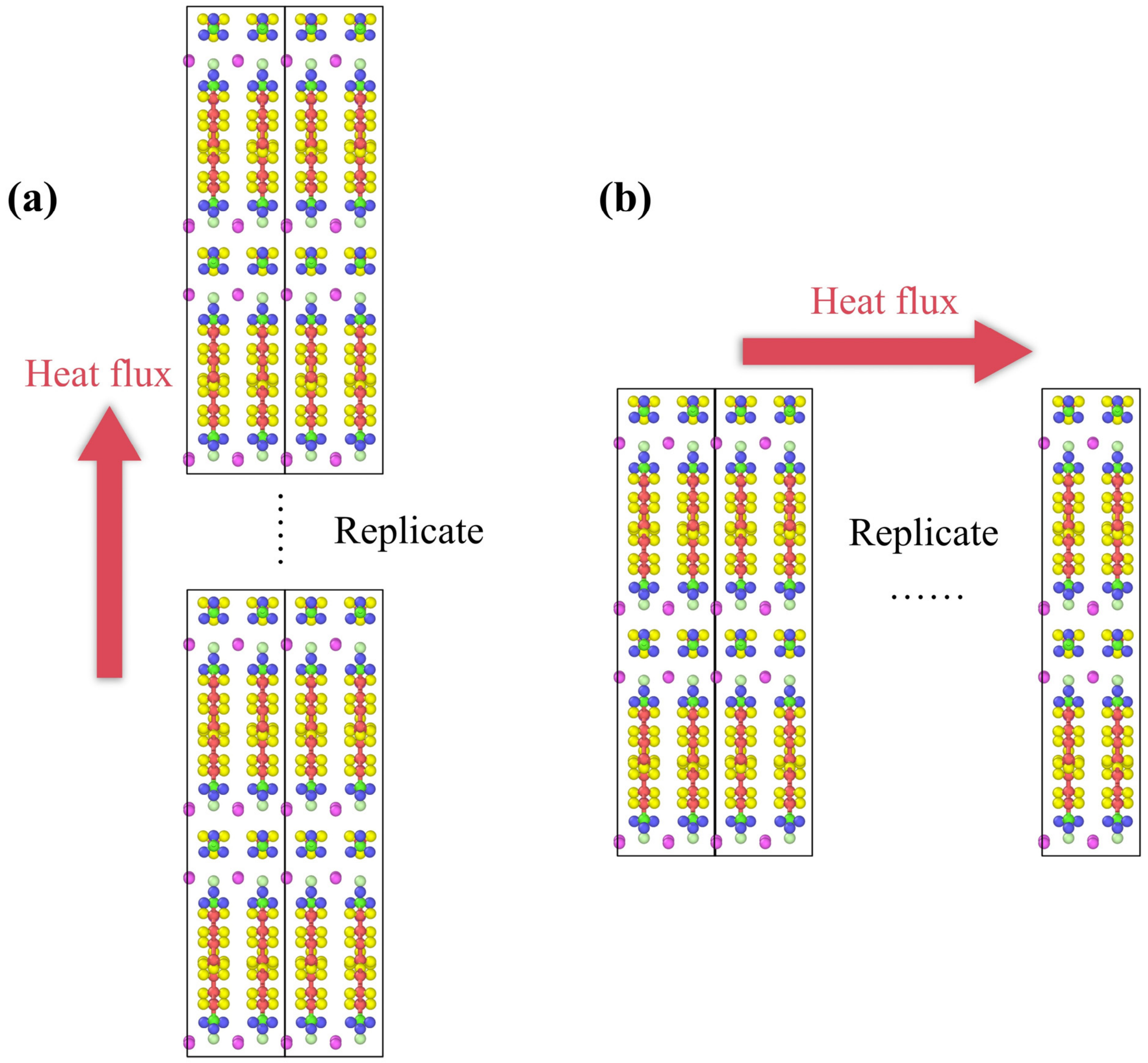

Figure S9. 2D perovskite with (a) parallel (b)perpendicular orientation and heat flux direction.

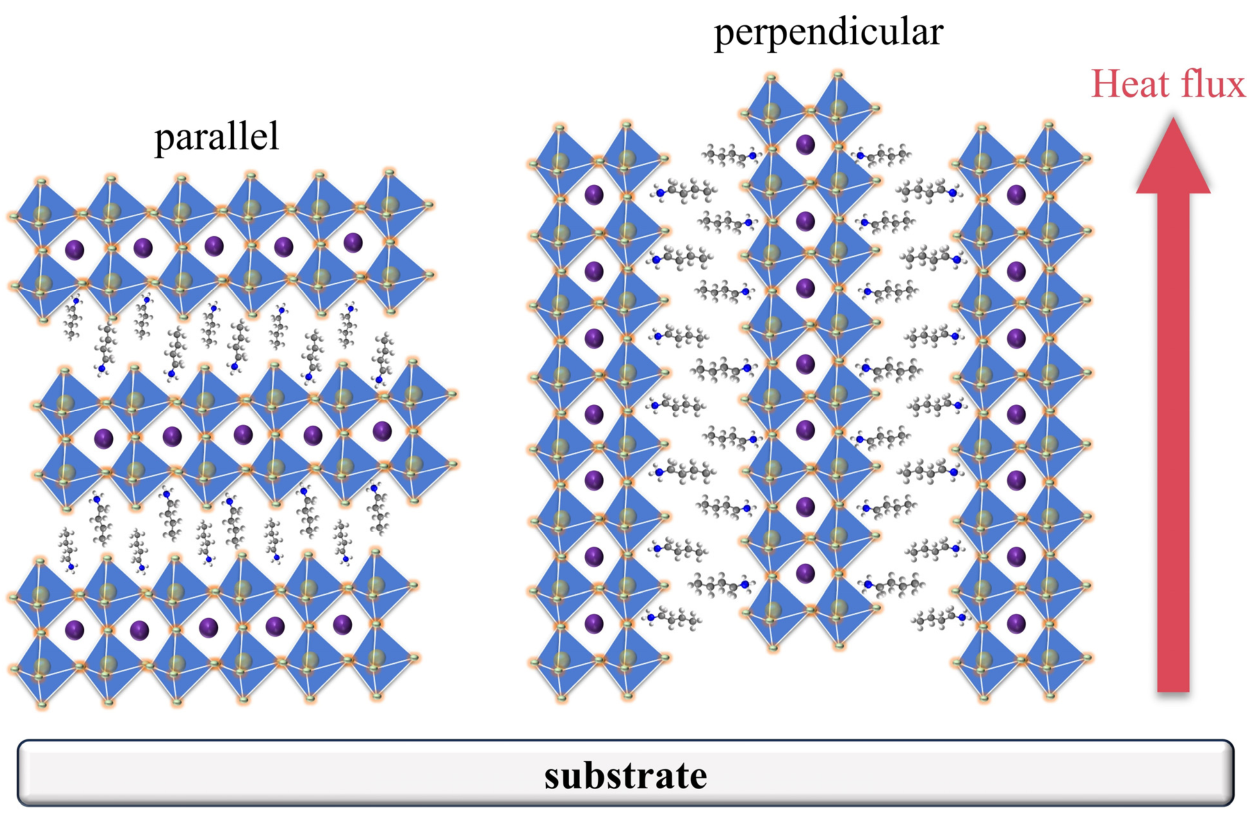

Figure S10. Diagram of 2D perovskite with (a) parallel (b) perpendicular orientation and heat flux direction.

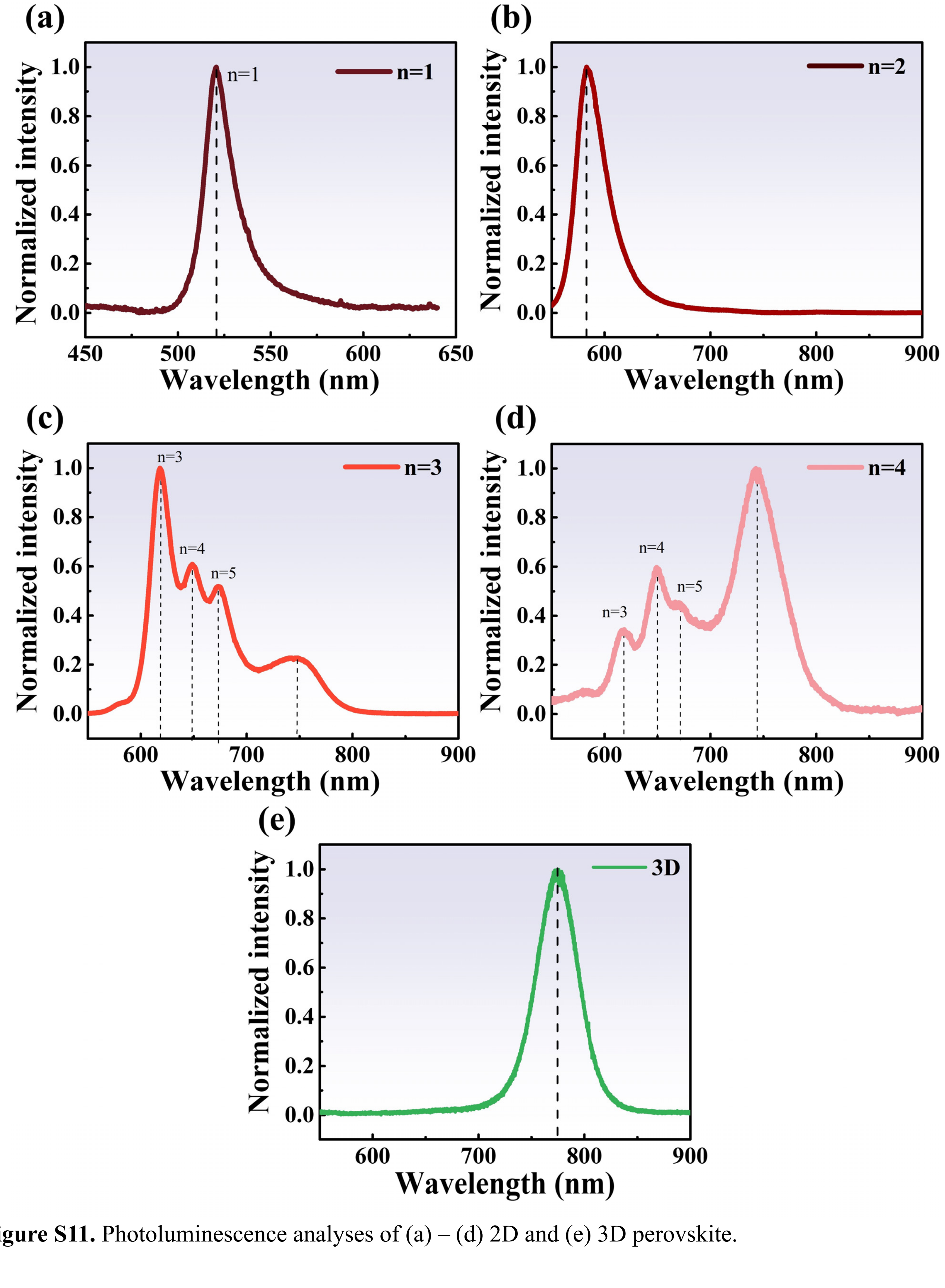

Figure S11. Photoluminescence analyses of (a) – (d) 2D and (e) 3D perovskite.
